# Nanoscale Structure of Lipid–Gemini Surfactant Mixed Monolayers Resolved with AFM and KPFM Microscopy

**DOI:** 10.3390/nano14070572

**Published:** 2024-03-26

**Authors:** Robert D. E. Henderson, Nanqin Mei, Yue Xu, Ravi Gaikwad, Shawn Wettig, Zoya Leonenko

**Affiliations:** 1 Department of Physics & Astronomy, University of Waterloo, Waterloo, ON N2L 3G1, Canada; robert.henderson@usask.ca (R.D.E.H.); nmei@uwaterloo.ca (N.M.); yue.xu@uwaterloo.ca (Y.X.);; 2 Waterloo Institute for Nanotechnology, University of Waterloo, Waterloo, ON N2L 3G1, Canada; 3 School of Pharmacy, University of Waterloo, Waterloo, ON N2L 3G1, Canada; 4 Department of Biology, University of Waterloo, Waterloo, ON N2L 3G1, Canada

**Keywords:** atomic force microscopy, Kelvin probe force microscopy, gemini surfactant, lipid monolayer, gene delivery system

## Abstract

Drug delivery vehicles composed of lipids and gemini surfactants (GS) are promising in gene therapy. Tuning the composition and properties of the delivery vehicle is important for the efficient load and delivery of DNA fragments (genes). In this paper, we studied novel gene delivery systems composed of 1,2-dioleoyl-*sn*-glycero-3-phosphocholine (DOPC), 1,2-dipalmitoyl-*sn*-3-phosphocholine (DPPC), and GS of the type N,N-bis(dimethylalkyl)-α,ω-alkanediammonium dibromide at different ratios. The nanoscale properties of the mixed DOPC–DPPC–GS monolayers on the surface of the gene delivery system were studied using atomic force microscopy (AFM) and Kelvin probe force microscopy (KPFM). We demonstrate that lipid–GS mixed monolayers result in the formation of nanoscale domains that vary in size, height, and electrical surface potential. We show that the presence of GS can impart significant changes to the domain topography and electrical surface potential compared to monolayers composed of lipids alone.

## 1. Introduction

Gemini surfactants (GSs) are a special class of synthetic amphiphilic molecules that are actively studied as candidates for gene delivery vectors. GS molecules possess two tails and heads, bound by a spacer group between their head groups [1,2]. These surfactants usually carry charges among their head groups, which allows them to interact with charged molecules or particles. Cationic GSs carry two positive charges, allowing them to interact with negatively charged DNA through electrostatic attractions [3,4,5,6,7,8,9,10].

With a judicious choice of spacers, heads, and tails, these systems can be very versatile with virtually no limit to their carrying capacity [7]. These systems have the potential to wrap and deliver packages of nanomaterials, with low toxicity, and are inexpensive to manufacture [7,11]. Currently, they are being studied as a means of efficiently packaging and transporting DNA, with a view to many biomedical applications, such as gene therapy [6,10,12,13,14,15,16].

In order to stabilize charged GSs and make them more compatible with cellular membranes, it is now common practice to add a ‘helper’ lipid to mixtures of GSs to facilitate cellular uptake [17,18,19,20], for example, 1,2-Dioleoyl-*sn*-glycero-3-phosphoethanolamine (DOPE) [12,20] and (DPPC) [21]. Electrostatic interactions between DNA and GSs are important for packaging DNA in nano-carriers and are dependent on the nanoscale properties of GS–lipid films. Thus, it is important to characterize the structure of these mixed GS–lipid films at the nanoscale to determine the most effective combination to construct the most efficient delivery carrier.

The two commonly used helper lipids DOPC and DPPC are the most studied in the phospholipid family [22,23,24,25,26]. These lipids, when mixed, are known to phase separate and form nanoscale domains, which differ by height and can be resolved using high resolution microscopy in bilayer or monolayer films [23,24,27]. These domains differ in shape and size, which depends on many factors: the ratios and the nature of the mixed phospholipids, their tail lengths [23,24], and the degree of saturation of the tails and the nature of the head groups. For example, DPPC is more ordered at 22 °C due to its higher melting temperature and is found in higher (more ordered) domains, while DOPC is in the fluid phase and is found in lower domains at this temperature [28]. These domains are important when the lipid membrane interacts with other biomolecules [29] and specifically when GS–lipid mixtures interact with DNA or other drug molecules to form a drug delivery system. While lipid model membranes or bilayers compose gene delivery vehicles, we used supported GS–lipid monolayers in this study, as lipid monolayers are widely accepted as excellent models to mimic complex model membranes and allow high-resolution imaging of nanoscale domains with AFM and KPFM [10,25,26,30,31,32,33,34,35].

**Figure 1:**
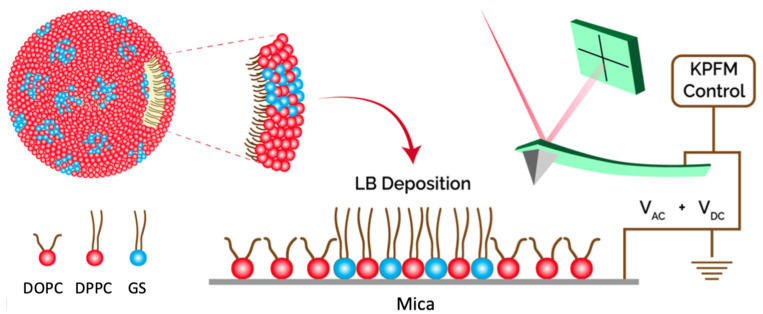
Schematic showing the spherical surface of the lipid–GS gene delivery system; the nanoscale domains formed in the monolayer due to phase separation in the DOPC–DPPC–GS mixture, shown in the upper surface of the sphere and in the supported monolayer set up for AFM and KPFM imaging.

GS monolayers suspended at air/water interfaces have been examined alone [36], as well as in the presence of fatty acids [37,38,39], DNA [4,10], or lipids [40]. Pure GS monolayers do show the formation of small domains at the air/water interface, suggesting some clustering in this environment [36]. When helper lipids are mixed with GS, the domains they form on the gene delivery systems are important for packing the DNA. In this work, we study nanoscale domains formed in mixed lipid (DOPC–DPPC) monolayers deposited on mica and how these domains are affected by the presence of GS molecules using AFM and KPFM imaging methods. Monolayer models serving as an analogue to the surface of a micelle or liposome drug delivery system were studied with microscopy methods when supported on a solid substrate (see Figure 1).

## 2. Materials and Methods

### 2.1. Overview of AFM and KPFM Methods

Atomic force microscopy (AFM) is a high-resolution imaging technique that has been widely used in biology and biophysics, specifically in membrane and protein studies [41,42,43]. In addition to AFM, which produces nanoscale resolution topography images, we used advanced Kelvin Probe Force Microscopy (KPFM) [44,45]. It combines the non-contact AFM with the Kelvin probe method, which allows the direct measurement of electrostatic properties at nanometer resolution in direct correlation to AFM topography images. While AFM is well established for biological applications, KPFM is commonly applied to study metallic and inorganic surfaces, but advanced modes of KPFM, such as amplitude modulation (AM) and frequency modulation (FM) [10,44,46,47], have been demonstrated to be useful for biological applications [29,30,41,48]. The operation principles of the amplitude modulation (AM) and frequency modulation (FM) [44,46], as well as details of the KPFM/AFM system used in this work are described in detail in Moores et al., 2010 [30], and in the Supplementary Materials.

In the present work, we used AFM and FM-KPFM to study the electrostatic and topographical domains in lipid–GS monolayers. The cationic nature of GSs is an ideal subject to advance the biological applications of KPFM in combined electrical surface potential topographical imaging. Surfactant molecules (lipid, GS, and other amphiphiles) all have dipole moments due to their inherent structure. When these dipole moments become aligned, as in a dry monolayer, they effectively become a sheet of dipoles, which gives rise to an electrostatic surface potential *V*, and the addition of charged molecules has a similar effect (see Equation (1)). KPFM is well suited to probe these features. We aim to show how GS behavior can be captured with these imaging techniques and thus used to assist the development of drug delivery research.

### 2.2. Lipids and Gemini Surfactants

For this study, we used a combination of 1,2-dioleoyl-*sn*-glycero-3-phosphocholine (DOPC) and 1,2-dipalmitoyl-*sn*-3-phosphocholine (DPPC), which is widely used as a simple model for cellular membranes (see the structure in the Supplementary Materials). DOPC and DPPC lipids were purchased from Avanti Polar Lipids Inc., https://avantilipids.com/. They were dissolved in stock 1 mg/mL solutions of chloroform that were kept in a freezer until use.

We used GSs of the type N,N-bis(dimethylalkyl)-α,ω-alkanediammonium dibromide, designated for simplicity as *m*-*s*-*m*, in which *m* is the length of the alkyl tails and *s* is the length of the spacer group in carbon atoms. We used GSs of two types: *m*-*s*-*m* = 12-3-12 and 16-3-16 in this work (see the structure in the Supplementary Materials). These were synthesized by reflux in acetone of the corresponding α,ω-dibromoalkane and N,N-dimethylalkylamine. After filtering and purifying by recrystallization, the chemical structures and purities were confirmed with NMR spectroscopy and surface tensiometry. For full details, see Wettig et al., 2007 [12]. The GS in powder form was stored in a desiccator and then dissolved in chloroform (at stock 1 mg/mL) before mixing with lipids. Lipids and GSs stock solutions were mixed at the ratio DOPC–DPPC–GS = 3:3:2 before spreading into an LB trough.

### 2.3. Langmuir–Blodgett Monolayer Deposition

**Figure 2:**
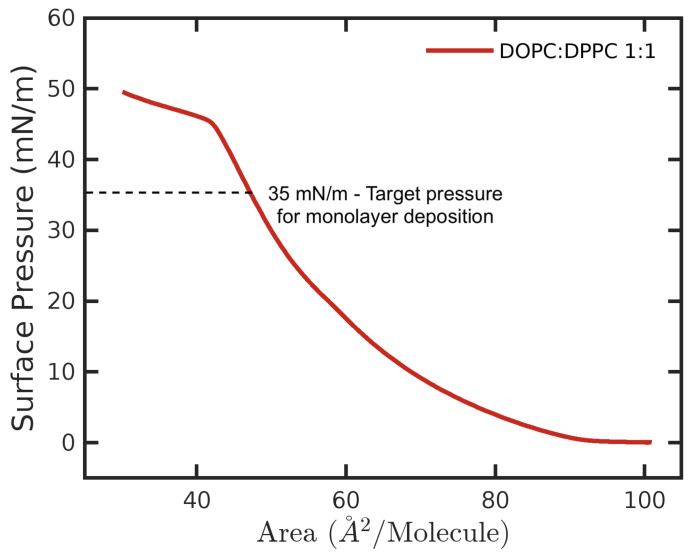
The averaged isotherm curve for DOPC–DPPC = 1:1 based on three repeated measurements. The dashed line indicates the position of 35 mN/m, which is the target pressure to deposit the lipid monolayer on mica. For more details, see the Supplementary Material Part III.

The GS–lipids monolayer is a useful model, as it forms the surface of the drug delivery system that interacts with the genetic material and is responsible for fusing with the cellular membrane. For AFM/KPFM imaging, monolayers were created using a Langmuir–Blodgett (LB) trough deposition as follows [49]. The LB trough was filled with water as the subphase, and the air/water interface was thoroughly cleaned so that compression yielded an increase in pressure of no more than 0.2 mN/m. Freshly-cleaved mica slides were suspended in the dipping well to rest just below the surface. The lipid–surfactant solution was deposited in several drops at the air/water interface to form a monolayer. The monolayer was left to equilibrate (and the chloroform was allowed to evaporate) for 10 min before compressing at 12 mm/min to a target pressure of 35 mN/m. This pressure was chosen to roughly correspond to cell membrane specifications and to allow phase contrast between the DOPC and DPPC lipids [50]. As shown in Figure 2, at the target surface pressure, the curve was in a smooth increasing region before reaching the left-end plateau, proving lipid monolayer formation at that surface pressure. The monolayer was deposited on the mica by raising the dipping arm slowly at a rate of 2 mm/min, while keeping the surface compressed at a constant pressure. The formed monolayer on mica is illustrated in Figure 1.

### 2.4. Imaging and Analysis

The AFM imaging was performed in contact mode using a JPK Nanowizard II and NCH-PPP cantilever tips with a resonant frequency near 150 kHz. FM-KPFM images were taken with an AIST-NT Smart SPM, using MikroMasch NSC-14-Cr/Au probes, with a resonant frequency of 130 kHz. In both cases, monolayer samples were deposited on mica slides and imaged in air. For resolving the electrical surface potential distribution in FM-KPFM mode, the supported monolayers on mica slides were placed onto conductive tape and connected to the KPFM electrode (Figure 1). Both AFM topography images and KPFM images were collected from the same sample area to allow a direct comparison between the topography and the electrical surface potential. Image analysis was performed using cross-section analysis and histogram methods with JPK image processing software (the details are described in the Supplementary Materials). For cross-section analysis, 100 measurements were collected from each sample to enable statistical analysis, and the surface coverage was analyzed using SPIP software. Analysis of the domain height difference (Δh) was performed using cross-section analysis and histogram methods. Analysis of the electrical surface potential distribution as contact potential difference (CPD = ΔV) was performed via analyzing cross sections of the raw unfiltered FM-KPFM images.

## 3. Results and Discussion

### 3.1. Results

#### 3.1.1. Monolayer Composition

A number of previous studies have shown that, when mixed, different phosphatidylcholine lipids (especially those with widely differing phase transition temperatures) form a monolayer composed of domains that exhibit a topographical contrast that is clearly shown in AFM images [50,51]. For this study, we used a DOPC–DPPC mixture, which is widely used as a simple model for cellular eukaryote membranes. In the mixed DOPC–DPPC bilayer, these lipids phase separate and form domains that differ in height [52]. The unsaturated acyl chains of the DOPC are more disordered (see the structures in the Supplementary Materials) and form domains that are approximately 1 nm shorter than DPPC domains, which yields a topographical contrast in AFM (higher and lower domains) [50]. The GSs are charged long-chain molecules (see the structures in the Supplementary Materials), and when they are mixed with DOPC–DPPC monolayers, we expect that they induce morphological changes in the domains that can be resolved by AFM topographical imaging. Next, we looked for changes in the electrostatic properties of the domains.

To identify the domains of DOPC and DPPC in our AFM images, we prepared control (no GS) monolayers composed of different ratios of DOPC to DPPC. As DPPC-enriched domains should be higher than DOPC [50], with the increase in the DPPC content, we should see a larger amount of area occupied by higher domains. For the effects of the GS, we added two different GSs to our lipid mixtures: *m*-*s*-*m* = 12-3-12 (GS-12) and 16-3-16 (GS-16), in a DPPC–GS molar ratio of 3:2, which has been found to be an appropriate ratio for effective gene transfection studies when helper lipids are used [53]. For more information about the experimental protocols and additional results, see the Supplementary Materials and the Ph.D. dissertation of Robert D. E. Henderson (see Ref. [49]).

#### 3.1.2. AFM Topography of the DOPC–DPPC Control Monolayer

**Figure 3:**
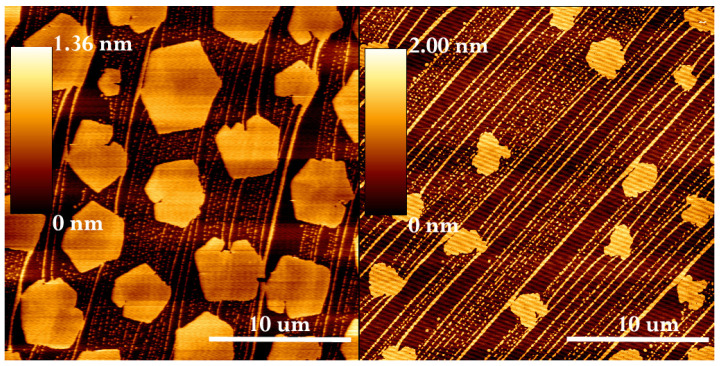
AFM topography images of control monolayers composed of lipids only: DOPC and DPPC in molar ratios of DOPC–DPPC 3:7 (**left**) and 1:1 (**right**) imaged in the air with the JPK NanoWizard instrument in contact mode.

Figure 3 shows the AFM topography images of the control monolayers (no GSs) containing different ratios of DOPC to DPPC. These lipid mixtures show a characteristic set of domains, with higher domains present in a series of circular or polygonal shapes in a matrix of lower domains. Frequently, there are notable streaks and striations aligned along a particular reference axis in the monolayer topography. This phenomenon has been reported previously [50,54] and is an artifact of the deposition that does not affect our results.

To confirm the identity of each lipid in higher or lower domains, we performed a surface analysis on a series of these images from random locations of each of the samples using the program Scanning Probe Image Processing (SPIP). We calculated the fraction of the surface that was covered by the higher domains (they appear lighter in the images) with two separate methods, and we show the results in Table 1. The first method used a histogram of pixel values, which takes advantage of the fact that our images were composed of domains at different heights. This appears in practice as two peaks in the histogram; the minimum between the two peaks roughly separated the domains, and we then calculated the fraction of the image occupied by the higher-height pixels (which thus calculated the surface coverage fraction of the higher domains). The second method employed an algorithm to identify particles (shapes) in the image, which were then summed to determine their surface coverage.

The results show that the surface coverage of the higher domains increased with the increasing DPPC proportion in the monolayer, indicating the higher domains were dominantly DPPC. From the nature of the structure of DPPC, at our deposition pressure (35 mN/m), it was in a gel-like phase, while the DOPC was in a fluid phase [55,56], suggesting the higher domains were enriched with DPPC, and the lower domains were enriched with DOPC (See details in the Experimental Section and the Supplementary Material Part III). These control samples were further characterized by cross-section analysis of the images shown in Figure 3. We found that, indeed, there was a strong correlation between the lipid ratio and the fraction of the surface occupied by the higher domains, with the details listed in Table 1. Specifically, at a 1:1 ratio of the lipids (by mol), 27(±4)% of the surface was covered by DPPC (higher domains), while 54(±4)% was covered at a ratio of 3:7 DOPC–DPPC. The compression isotherm of 1:1 DOPC–DPPC can be found in the Supplementary Materials.

#### 3.1.3. AFM/KPFM Images of DOPC–DPPC Monolayers with Gemini Surfactant

**Figure 4:**
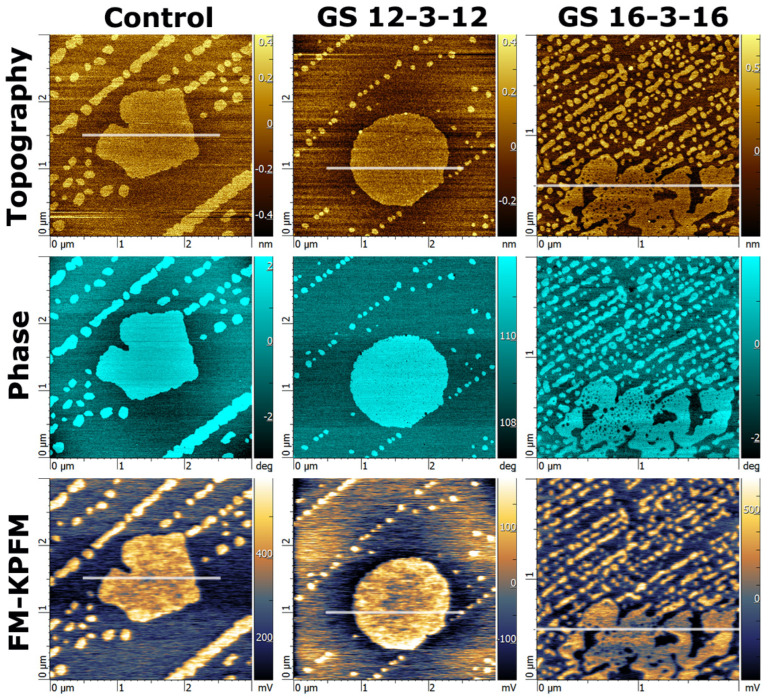
AFM and KPFM images of the DOPC–DPPC supported monolayers with and without GS: first raw AFM topography images, second AFM phase images, and third KPFM electrical surface potential images.

**Figure 5:**
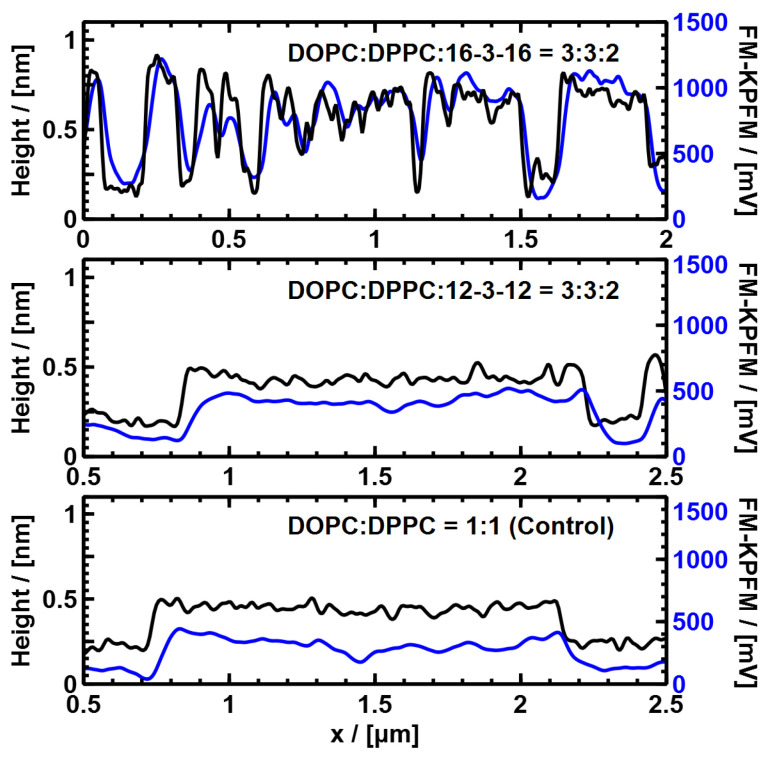
Cross-section plots showing the height variation and electrical surface potential variations along the line shown in the AFM and KPFM images in Figure 4.

Figure 4 and Figure 5 show AFM and FM-KPFM images of the samples containing DOPC–DPPC with the GS-12 and GS-16 and a control (DOPC–DPPC with no GS) sample. The addition of GS-12 had little or no effect on the DPPC domain structures. However, when GS-16 was added, an intricate domain formation was observed, which indicates the incorporation of GS-16 into the lipid monolayer.

A combined cross-section analysis was performed to determine the height difference between domains (Δh) and the electrical surface potential difference (ΔV) (see the Supplementary Materials for details). The results of this analysis are shown in Table 2. For the DOPC–DPPC control monolayer, the Δh between the domains (DPPC and DOPC) was 0.33 ± 0.01 nm, and ΔV was 336 ± 7 mV. The Δh in our results was somewhat lower than other studies near our deposition pressure (35 mN/m) [50]. With the addition of GS-16, the height difference (Δh) increased to 0.57 nm (0.57 ± 0.01 nm), with a ΔV increase to 658 ± 17 mV. This indicates that GS-16 interacted with the DPPC and DOPC lipids differently, and the increased positive ΔV between the domains was consistent with adding cationic molecules to the higher DPPC domains. The addition of GS-12 resulted in very small changes with a height difference (Δh) decrease to 0.28 ± 0.02 nm and a ΔV decrease to 304 ± 13 mV.

Figure 4 and Figure 5 show that the control monolayer and the monolayer with GS-12 had similar domain widths in the x–y plane. Both samples possessed a large domain with regular margins surrounded by sporadic smaller domains. In contrast, the GS-16–lipid monolayer exhibited wider irregular domains, which look like a large amount of smaller domains and joint coagulation of smaller domains in a larger structure. Moreover, the sporadic smaller domains in this monolayer existed in a higher frequency, resulting in increased overall surface coverage. This observation is consistent with the previous report that suggested the GS-12 does not incorporate well into lipid monolayers, likely due to its high preference for dissolving in the subphase solution, whereas the GS-16 exhibits higher affinity with phospholipids [57,58]. Another contributing factor could be the shorter chain length and higher solubility in water of the GS-12 compared to the longer-chain GS-16 [59].

### 3.2. Discussion

The maps of the electrical surface potential revealed by the KPFM images in our samples can be explained by considering the dipole nature of the lipids. As the simple uniform monolayer has an electrical surface potential *V*, given by Brockman and Howard [60] as

(1) $$V=12\pi \frac{\mu_{⊥}}{A},$$

in which $\mu_{⊥}$ is the normal component of the dipole moment of each molecule in MDebye, *A* is the area per molecule in Å^2^, and *V* is measured in mV. This is valid with the assumption that the sheet of dipoles is infinite in area. However, real monolayers, and especially monolayers composed of mixed lipids, have a finite total area and form domains of different molecules or similar molecules at different orientations or densities; thus, this equation would only be a reasonable approximation for either small scanning heights (i.e., near contact) and/or for large domains. Many factors contribute to the resulting dipole moments of the DOPC and DPPC monolayers: the dipole of a single lipid molecule; packing density of the lipids; lipid orientation with respect to the surface (tilt); as well as the scanning probe itself. Due to the last factor (scanning probe), KPFM analysis can in principle only lead to knowledge of the relative electrical surface potentials (ΔV), and a determination of an absolute potential can only be performed by other methods [61] that are beyond the scope of this work. In our experiments, the same scanning probe was used; thus, the differences in height and *V* can be reliably measured and compared.

It is not straightforward to illustrate the precise relationship between an experiment and theory; however, here, a simplified analysis guided by previous publications is provided [33,60,62,63,64,65]. Given that the hydrophilic mica slides are pulled out of the aqueous subphase during the deposition process, the lipid and surfactant molecules will have their head groups oriented toward the substrate, with the hydrophobic tails on the visible surface of the samples. Previous studies have shown that the total dipole moments of phospholipids are positive toward the air [60,66,67,68]. A literature review on this topic shows the dependence of properties such as the dipole moment or electrical surface potential on conditions such as the surface pressure, molecular area, and subphase composition for DOPC and DPPC. Table 3 presents the results of this review. While the picture is by no means complete, we can glean useful quantities from these data and relate them to our present measurements.

Given the surface analysis results and the values for the molecular areas shown in Table 3, it is obvious that the molecular density of DPPC is higher than DOPC; thus, DPPC will have a boost to higher values for the electrical surface potential relative to DOPC. This is because the area per molecule is smaller for DPPC than DOPC at the same pressure (shown in Table 3). This is in fact what is shown for the monolayers in Figure 4 and Figure 5. In addition, noting that DPPC was measured to be higher than DOPC, despite DPPC having a similar carbon chain length [28], we must conclude that the fluid-like nature of DOPC causes it to be more disordered and oriented non-perpendicularly to the substrate surface. Therefore, the component of the dipoles of DOPC that is normal to the mica substrate will be smaller than DPPC (at a similar density). A combination of these two factors (density and orientation) must therefore contribute to the difference in the electrical surface potential between the two lipid domains. Ultimately, the ΔV between DOPC and DPPC is in line with the values presented in the studies shown in Table 3, in which, for example, at a pressure of 30 mN/m the difference was given to be 271 mV. The trend that is observed for the pressure dependence of the potential suggests that the result would be slightly higher for a higher pressure (35 mN/m in our experiments), which is consistent with our results, ΔV = 336 mV.

**Table 3:** A summary of the literature values for the area per lipid (*A*), dipole moments ($\mu_{⊥}$), and the electrical surface potential (*V*) of pure DOPC and DPPC monolayers at various surface pressures (π). The calculated theoretical differences in the electrical surface potential (ΔV) between DPPC and DOPC, based on the literature values, are shown at the bottom of this table, in comparison with the experimental value obtained in this work.

π	A	V	$\mu_{⊥}$	Subphase	References
(mN/m)	(Å^2^)	(mV)	(mD)		
DOPC					
20	78	306	632	H_2_O	[66]
30	70	329	610	H_2_O	[66]
35	70	no data	850	NaCl ^*a*^, pH 6	[68]
45	59	311 ^*e*^	486	H_2_O	[67]
45	58	no data	463	PBS ^*b*^, pH 6.6	[67]
no data ^*c*^	no data	384	no data	KCl ^*d*^	[69]
DPPC					
10	69	no data	469	PBS ^*b*^, pH 6.6	[67]
20	46	400	488 ^*e*^	H_2_O	[63]
30	44	600	700 ^*e*^	H_2_O	[63]
35	42	700	780 ^*e*^	H_2_O	[63]
35	45	no data	700	NaCl ^*a*^, pH 6	[68]
no data ^*c*^	no data	460	no data	KCl ^*d*^	[69]
23	no data	640	no data	Simulation	[34]
Differences in Electrical Surface Potential: $\Delta V=V_{\mathrm{DPPC}}-V_{\mathrm{DOPC}}$					
20		94		H_2_O	Calculated from data above
30		271		H_2_O	Calculated from data above
35		336		H_2_O ^*f*^	**This work**

^*a*^ Subphase 0.1 M NaCl. ^*b*^ Subphase 0.01 M phosphate buffer with 0.1 M NaCl. ^*c*^ Lipids added until *V* stopped changing. See Ref. [69] for details. ^*d*^ Subphase 1 mM KCl. ^*e*^ Computed from Equation (1). ^*f*^ Supported monolayer on mica prepared with LB deposition using a pure water subphase.

When GS-16 was added to the DOPC–DPPC monolayer, the electrical surface potential difference was 658 mV and was much larger compared to the control monolayer (336 mV). Given the lack of analogous potential and dipole data for these GSs compared with the common lipids, for the present purpose, if we make the simplifying assumption that the only *new* contribution to the surface potential is due to the 2+ charges on the surfactant molecules (that is, to assume that the dipole moments of DPPC are the same as the dipole moments of GS-16 without the 2+ charge), the molecular density of GS that has integrated into the DPPC domains can be computed. Assuming also that the sizes of the domains are much larger than the tip-sample separation during the scan so that we may take the distribution of surfactant molecules to be an infinite sheet of charge, we have

(2) $$V_{e^{-}\;\mathrm{sheet}}=\frac{z\sigma }{2\epsilon_{0}},$$

in which *z* is the height of the tip during the measurement and σ is the surface charge density. The density of the GS molecules is then

(3) $$\sigma_{\mathrm{gemini}}=\frac{\epsilon V_{e^{-}\;\mathrm{sheet}}}{z},$$

since there is a charge of 2+ on each molecule. Making the further assumption that the area per molecule is the same within the DPPC domains infused with GS, as with the DPPC alone (45 Å^2^), this calculates to about one GS molecule per four molecules of DPPC, as shown by Drolle et al. in 2017 [41]. This is a plausible result (consistent by order of magnitude), given the assumptions and our starting relative concentrations of 3:2 for DPPC–GS.

## 4. Conclusions

We studied the effects of GS on DOPC–DPPC monolayers using AFM and KPFM imaging. We resolved topographical and electrostatic domains in mixed DOPC–DPPC monolayers that were little affected by the inclusion of GS-12, as both the differences in the domain heights and the electrical surface potential were statistically consistent with the DOPC–DPPC monolayer. With the use of GS-16, we observed a stronger interaction between the GS and DPPC, with more intricate domain formation, and a much more positive electrical surface potential difference (ΔV changed from 336 mV in the DOPC–DPPC monolayer to 658 mV in the GS–DOPC–DPPC monolayer. Mixing GS with DOPC–DPPC lipid produced a stable monolayer with nanoscale domains and more positive electrical surface potential than the control monolayer without GS.

While the morphology of these domains in the monolayer mixtures can be determined via AFM, the unique capabilities of KPFM provide valuable information on electrostatic properties and are especially useful in studying the properties of the lipid monolayers in the presence of the cationic GS. Future development of efficient molecular delivery vehicles based on these mixtures requires a detailed nanoscale characterization of the monolayers they form and how they behave in different environments. Characterization of electrostatics, composition, and morphology on the nanoscale will help to develop a theoretical and biophysical framework for these new systems. Future work by our group will explore a wider variety of these systems containing an array of GSs and phospholipids. 

## Figures and Tables

**Table 1:** Surface coverage analysis for higher domains in the control samples at two ratios of DOPC–DPPC. The results are shown for both the histogram and particle detection surface coverage analysis of higher domains, averaged over four experiments, which are described in the Supplementary Materials. All numerical values in this table are expressed as percentages.

DOPC–DPPC	Higher Domain Surface Coverage		Model Average
(mol)	(Histogram)	(Particle)	
1:1	29 ± 3	25 ± 3	27 ± 4
3:7	55.6 ± 0.6	53 ± 4	54 ± 4

**Table 2:** Results of the image analysis collected from the FM-KPFM electrical surface potential images and AFM topography images, where differences between the domains were calculated and denoted as ΔV and Δh using the cross-section method. The lipid monolayers were the control (DOPC–DPPC = 1:1), with GS-12 (DOPC–DPPC–GS-12 = 3:3:2), and with GS-16 (DOPC–DPPC–GS-16 = 3:3:2). See the text and Supplementary Materials for further details.

	FM-KPFM (ΔV)	AFM (Δh)
DOPC–DPPC (Control)	336 ± 7 mV	0.33 ± 0.01 nm
DOPC–DPPC–GS-12	304 ± 13 mV	0.28 ± 0.02 nm
DOPC–DPPC–GS-16	658 ± 17 mV	0.57 ± 0.01 nm

Margins of error calculated at a 95% confidence level.

## Data Availability

Data are contained within the article.

## Supplementary Materials

The following supporting information can be downloaded at: https://www.mdpi.com/article/10.3390/nano14070572/s1. Molecular structure of the lipids and GSs; compression isotherm of 1:1 DOPC and DPPC; principle of AFM and FM-KPFM methods; histogram and cross-section analysis of AFM and FM-KPFM images. References [10,30,41,44,46,49,70,71,72,73,74,75,76,77,78] are cited in the Supplementary Materials.
