# Supplementary material for: Nanoscale Structure of Lipid–Gemini Surfactant Mixed Monolayers Resolved with AFM and KPFM Microscopy
Source: Nanomaterials (Basel). 2024 Mar 26;14(7):572. doi: 10.3390/nano14070572 (PMC11013119; doi:10.3390/nano14070572)
Supplement: Supplementary file 1 [file nanomaterials-14-00572-s001.zip › nanomaterials-2827401-supplementary.pdf]

Nanoscale Structure of Lipid–Gemini Surfactant Mixed Monolayers Resolved with AFM and KPFM Microscopy – Supplementary Material

Robert D. E. Henderson,[a,b] Nanqin Mei,[a,b] Yue Xu,[a]
Ravi Gaikwad,[a] Shawn Wettig,[b,c] and Zoya Leonenko*[a,b,d]

E-mail: zleonenk@uwaterloo.ca
[a]Department of Physics & Astronomy, [b]Waterloo Institute for Nanotechnology,
[c]School of Pharmacy and [d]Department of Biology,
University of Waterloo, Waterloo, ON N2L 3G1, Canada

I. MOLECULAR STRUCTURES OF THE LIPIDS AND GEMINI SURFACTANTS

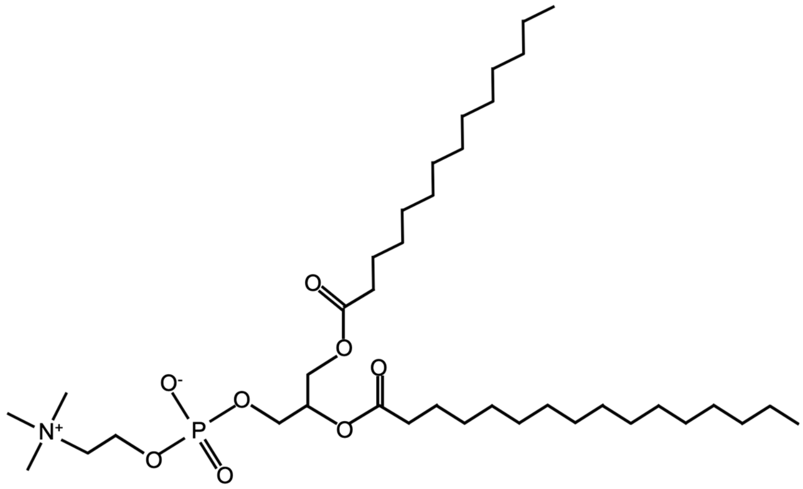

Figure S1. Molecular structure of 1,2-dipalmitoyl-sn-3-phosphocholine (DPPC).

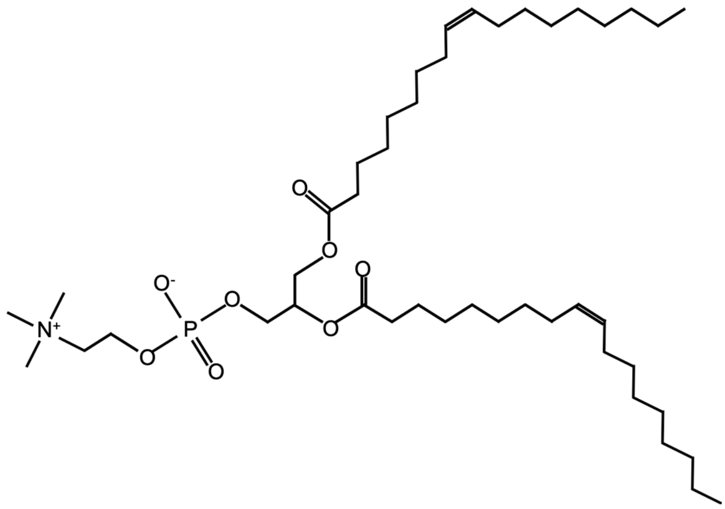

Figure S2. Molecular structure of 1,2-dioleoyl-sn-glycero-3-phosphocholine (DOPC).

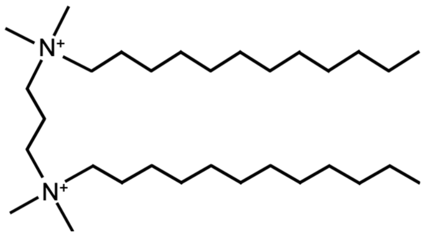

Figure S3. Molecular structure of gemini surfactant 12-3-12 (GS-12).

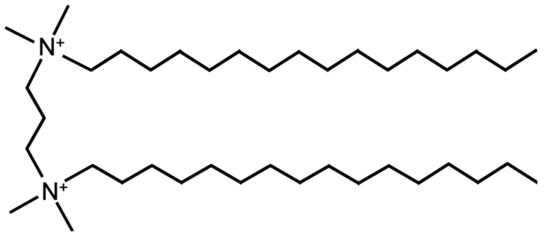

Figure S4. Molecular structure of gemini surfactant 16-3-16 (GS-16).

II. PRINCIPLE OF AFM AND FM-KPFM METHODS

Atomic force microscopy (AFM) is a scanning probe microscopy technique, where images are produced by scanning the sample surface with a micro-machined cantilever with an attached sharp scanning probe or tip. The physical interactions between the probe and the sample are recorded at each point to construct a 3D image[1]. AFM imaging can be performed in three modes: contact, tapping (intermediate), or non-contact mode[2].

Kelvin Probe Force Microscopy (KPFM)[3] is a type of scanning probe microscopy, which combines the non-contact AFM with the Kelvin probe method[4], to allow a direct measurement of electrostatic properties at nanometer resolution. The operation schematic of a KPFM system is shown in Figure 1 of the main text. In a KPFM measurement, a conductive cantilever (AFM cantilevers that are coated with Pb or Au, etc.) is brought into electrical contact with the sample surface, allowing the formation of a vibrating capacitor (C) between the tip and the sample, such that the electrostatic force (F_{el}) is used as the controlling parameter in the feedback loop, generally expressed as Equation. 1,

$$F_{el} = -\frac{1}{2}\frac{dC(z)}{dz}V^2, \tag{1}$$

where z is the vertical between the tip and the sample and V is the total voltage applied to this capacitor. In addition to the applied AC voltage ($V_{AC}\sin(\omega_{AC}t)$) to generate cantilever oscillation in the non-contact AFM, KPFM requires an extra applied DC voltage (V_{DC}) to the cantilever. As in the Kelvin method[4], the vibrating capacitor generates an alternating current if there is a contact potential difference (CPD) between the plates. Normally, the V_{CPD} is generated from the difference

between the work functions of the capacitor plates or any electrical surface potential that is present ($V_{CPD} = (\phi_{sample} - \phi_{tip})/e$), affecting the AFM amplitude response. Therefore, a bias voltage V_{DC} is applied to compensate for the effect of V_{CPD}. In the KPFM technique, the AC voltage, namely modulation voltage, acts in combination with this vibrating capacitor system and links the contact potential difference with the bias voltage via the modulation frequency ω_{AC}. At this point, Equation 1 can be further expressed as the following equation,

$$F_{el}(z,t) = -\frac{1}{2}[(V_{DC} \pm V_{CPD}) + V_{AC}\sin(\omega_{AC}t)]^2 \frac{\delta C(z)}{\delta z}. \quad (2)$$

Expanding the right side of Equation 2, one could derive F_{el} into the following equation

$$F_{el}(z,t) = -\frac{1}{2}\{(V_{DC} \pm V_{CPD})^2 + 2(V_{DC} \pm V_{CPD})V_{AC}\sin(\omega_{AC}t) + \frac{1}{2}{V_{AC}}^2[cos2(\omega_{AC}t) - 1]\}\frac{\delta C(z)}{\delta z}, \quad (3)$$

where the middle term generates a signal of alternative force $F_{\omega_{AC}}$, and is used for CPD detection (or work function in the case of metals):

$$F_{\omega_{AC}} = -(V_{DC} \pm V_{CPD})V_{AC}\sin(\omega_{AC}t)\frac{\delta C(z)}{\delta z}. \quad (4)$$

The remaining two terms in Equation 3 are then rearranged to give F_{DC} and $F_{\omega_{AC}}$,

$$F_{DC} = -[\frac{1}{2}(V_{DC} \pm V_{CPD})^2 + \frac{{V_{AC}}^2}{4}]\frac{\delta C(z)}{\delta z}, \quad (5)$$

$$F_{2\omega_{AC}} = \frac{{V_{AC}}^2}{4}\cos(2\omega_{AC}t)\frac{\delta C(z)}{\delta z}, \quad (6)$$

where F_{DC} contributes to the topographic signal and $F_{2\omega_{AC}}$ contains the signal at $2\omega_{AC}$ that is used for capacitance microscopy.

Changes in the tip-sample interaction cause changes in the amplitude of the tip oscillation, which provides a mechanism for a feedback loop to record the CPD during the scanning procedure[5]. In the most basic operating mode, the topography is recorded first in the standard AFM way and using that information a second scan is performed when the cantilever hovers at a constant height (~ 50 nm, where the interaction is mostly electrostatic in origin) to record the CPD as described above, which is referred to as lift mode.

Two specialized KPFM modes offer significant improvements on the basic mode: amplitude modulation (AM) and frequency modulation (FM)[3,6]. In AM mode, the cantilever is driven to oscillation at a resonant frequency, measuring the sample height and CPD. Therefore, a resonance-enhanced detection is realized, enabling high sensitivity to electrostatic force while keeping ω_{AC} low. A frequent way to achieve the measurement is the two-pass way using lift mode, as described in the above text. To further enhance the CPD signal, a much higher resonant frequency, i.e. the second oscillation mode, is chosen instead of the fundamental value that is used for topographic measurement during the first scan pass. This enhancement amplifies the oscillation at ω_{AC} by a factor of Q (quality factor), while allowing the modulation voltage down to 100 mV level. However, the fact that the second resonance frequency is usually about six times higher than the fundamental value limits the AM mode, due to the detection boundary of the photodiode.

FM mode is similar to AM mode, except that the system tracks a signal that arises from the oscillation of the electric force gradient that is due to the modulation voltage. The proportionality between the measured frequency shift and the electric force gradient is shown in the following equations,

$$\Delta f \propto \frac{\delta F_{\omega_{AC}}}{\delta z}, \quad (7)$$

substitution of Equation 4 into Equation 7 gives

$$\Delta f \propto (V_{DC} \pm V_{CPD})V_{AC} \sin(\omega_{AC} t) \frac{\delta^2 C(z)}{\delta^2 z}, \quad (8)$$

here ω_{AC} is chosen to be only a few kHz. Since the fundamental resonance frequency of a cantilever is shifted due to a force gradient, oscillations in the force gradient generate side-band signals due to frequency mixing. A KPFM system can track signals at these side bands in a feedback loop that nullifies them. A null signal at these side bands indicates a compensated, and thus recorded, CPD. Since the force gradient is large near the sample surface, and not subject to any parasitic influence from the topography, FM-KPFM can achieve the greatest spatial resolution in air. For further details on the technical specifics of KPFM and these modes, see Refs. 3,5–7.

III. COMPRESSION ISOTHERM OF 1:1 DOPC:DPPC LIPID MODEL

The compression isotherm experiments for the DOPC:DPPC model in a molar ratio of 1:1 were conducted at 22.5 °C. The concentration of the lipid mixture solution in chloroform was 1 mg/mL. The compression speed of barriers was 12 mm/min, consistent with the speed used for monolayer deposition. Three repeats were finished to indicate the reproducibility of the DOPC:DPPC isotherm curve, as indicated in Figure S5. The reason that we chose 35 mN/m as indicated in Figure S6 for monolayer deposition is to mimic the natural state of biological membranes, which usually lies between 30 mN/m and 35 mN/m as confirmed by previous studies[8-11] and as we previously used in our lab[12, 13].

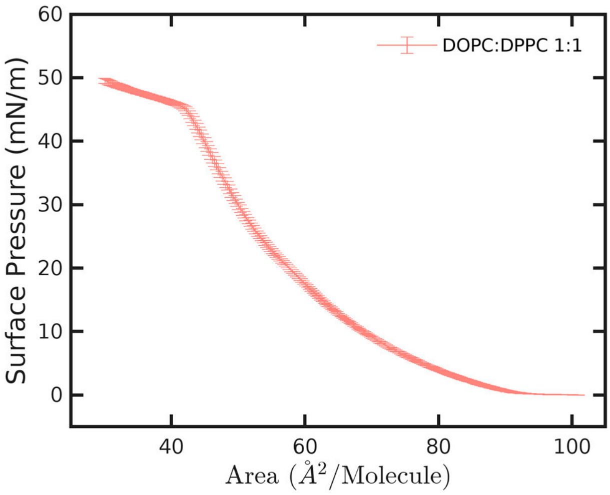

Figure S5. The averaged isotherm for DOPC:DPPC = 1:1 based on three repeated measurements including error bar.

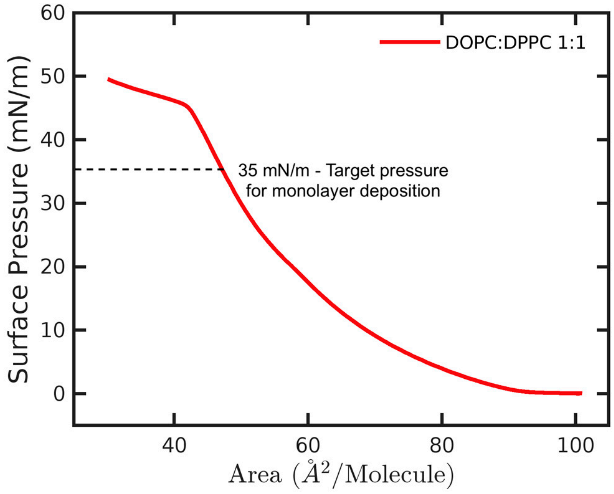

Figure S6: The averaged isotherm curve for DOPC:DPPC = 1:1 based on three repeated measurements without error bar. The dash line indicates the position of 35 mN/m, which is the target pressure to deposit lipid monolayer on mica.

IV. HISTOGRAM AND CROSS-SECTION ANALYSIS OF AFM AND FM-KPFM IMAGES

Table 1: Examples of image analysis results for three methods to compute height and surface potential differences between domains in lipid monolayers: control (DOPC+DPPC 1:1) and with GS-16 (DOPC+DPPC+GS-16 3:3:2). Values for the histogram method were derived from 5 sectors from each image. For masking, one mask was created for each image of both the domains and background to calculate the difference. Fifty individual measurements were used for each of the cross-section values. The cross-section method was used to calculate result for the height and surface potential of the domains relative to the background.

Method	Height / (nm)				FM-KPFM / (mV)			
	Trial 1		Trial 2		Trial 1		Trial 2	
	$\bar{x}_{1,H}$[a]	$s_{1,H}$[b]	$\bar{x}_{2,H}$	$s_{2,H}$	$\bar{x}_{1,K}$	$s_{1,K}$	$\bar{x}_{2,K}$	$s_{2,K}$
	DOPC:DPPC 1:1 (Control) Monolayer							
Histogram	0.274	0.018	0.262	0.017	286.2	31.3	314.0	45.2
Masking	0.276	0.089	0.309	0.085	261.6	64.4	318.8	46.7
**Cross Sections**	0.328	0.045	0.335	0.043	339.7	40.4	332.6	33.1
	DOPC+DPPC With GS-16							
Histogram	0.567	0.026	0.559	0.043	597.8	69.8	567.4	47.1
Masking	0.621	0.104	0.587	0.099	705.0	144.8	632.0	116.5
**Cross Sections**	0.582	0.047	0.566	0.073	654.3	91.5	660.7	75.0
Combined Cross Section Data[c]								
Control	0.33 ±0.01 nm				336 ±7 mV			
With GS-16	0.57 ±0.01 nm				658 ±17 mV			

[a] Sample mean, $\bar{x}_i$, of trial i, with H (height) or K (FM-KPFM).

[b] Standard deviation of trial.

[c] Margins of error calculated at a 95% confidence level.

In this section, we describe three possible methods by which to analyze these images but conclude that a direct cross-section analysis provides the most plausible result. Details of the methods are presented in the PhD dissertation from Robert. D. E. Henderson (see Ref. 14). The first method is to use histograms of the pixel values, in much the same way that was done for the surface coverage analysis. In this case, however, instead of using the histograms to calculate a fraction of the total number of pixels, we can use them to determine the difference in the height or surface potential between the domains. Suppose we have, in an ideal situation, a clean background of some height h_b, with Gaussian noise of relatively small standard deviation. Now add domains of height h_d, again with Gaussian-distributed noise with a standard deviation much smaller than $h_d - h_b$. A histogram of these pixel values will show two Gaussian peaks, separated by $h_d - h_b$. We employ this procedure on two high-quality images of each experiment. To mitigate intra-image variation (e.g., flattening effects, artefacts), we draw 5 histograms from each image that were taken from different sub-regions representing good quality domains with the background. The result is then averaged for each image (each trial).

A second method is to use the freely available program *GWYDDION*[15] to extract image statistics. While the algorithms are not as advanced as SPIP, it is straightforward to 'paint' a mask onto each image to cover the region of interest and compute statistical quantities of the masked pixels. An alternative method is to simply threshold an image; that is, to select all pixels above or below a threshold value. In most cases, this will suffice to pick out a reasonable amount of background or domains. In general, to avoid edge effects, it is necessary to 'shrink' the masks to focus on the central regions of the domains or background. This was done for the same images as the histogram method above, and the mean and standard deviation of the pixel values were obtained to compute the difference in height and FM-KPFM signal.

Another method is more direct: one may take a series of cross-sections within the image and manually select background and domain regions from which to compute height and surface potential differences. While this is more labor-intensive and can be subjective if one is not careful, overall, it is a very reliable method as it allows direct confirmation of the measurements.
The AIST-NT image analysis software allows semi-automatic computation of differences within the cross-sections by selections of peaks and valleys. For each of our images, 100 measurements were taken in this manner, and their means and standard deviations were obtained. In most cases, a small amount of gaussian smoothing applied to the images greatly enhanced the quality.

An analysis of the data confirmed that the two trials for each of the histogram and cross-section methods, for each experiment, yielded results that were statistically consistent between the trials (but not with each other). However, the masking method gave inconsistent values for the means from each trial in all cases. Therefore, we reject the masking method for its unreliability. Summaries of these data are given in Table 1 in the end of this supplementary material. We note that the histogram method produces values that are consistently below those obtained from the

direct cross-section measurements. This can be explained as follows: the histogram peaks from which we measure the difference gives us the difference between the two most frequent pixel values, assuming a roughly Gaussian distribution for each. The locations of these peaks are sensitive to edge effects of the interfaces between the domains and the background and to any variation in the calibration (flattening) across the image. It is also true that in our ideal case described above the histogram method would yield acceptable results, but these two issues create problems. The most frequent pixel values are not necessarily those of the top surface of the domains, and this problem is inflated when the domains are not very large compared to the size of the pixels. For these reasons, the histogram method consistently underestimates the height and FM-KPFM differences. Therefore, we turn to the cross-section method from here on.

The use of cross sections to measure differences between the domains and the background is sound for a few reasons. Firstly, the method shows itself to be statistically consistent from image to image. Second, each line is not subject to global variations in the image calibration since the measurements are taken from a single scan line. And third, many measurements can be obtained on a single image and the quality of the data is confirmed by the experimenter.

References

1. Martin, Y.; Williams, C.C.; Wickramasinghe, H.K. Atomic force microscope–force mapping and profiling on a sub 100-Å scale. J. Appl. Phys. 1987, 61, 10135–10139.
2. Jalili, N.; Laxminarayana, K. A review of atomic force microscopy imaging systems: application to molecular metrology and biological sciences. Mechatronics 2004, 14, 907–945.
3. Sadewasser, S.; Glatzel, T. Kelvin Probe Force Microscopy; Springer: Berlin/Heidelberg, Germany, 2012.
4. Kelvin, L. V. Contact electricity of metals. London Edinburgh Philos. Mag. J. Sci. 1898, 46, 82–120.
5. Melitz, W.; Shen, J.; Kummel, A.C.; Lee, S. Kelvin probe force microscopy and its application. Surf. Sci. Rep. 2011, 66, 1–27.
6. Zerweck, U.; Loppacher, C.; Otto, T.; Grafström, S.; Eng, L.M. Accuracy and resolution limits of Kelvin probe force microscopy. Phys. Rev. B 2005, 71, 125424. https://doi.org/10.1103/PhysRevB.71.125424.
7. Moores, B.; Hane, F.; Eng, L.; Leonenko, Z. Kelvin probe force microscopy in application to biomolecular films: frequency modulation, amplitude modulation, and lift mode. Ultramicroscopy 2010, 110, 708–711. https://doi.org/10.1016/j.ultramic.2010.02.036.
8. Janmey, P.A.; Kinnunen, P.K.J. Biophysical properties of lipids and dynamic membranes. Trends Cell Biol. 2006, 16, 538-546. https://doi.org/10.1016/j.tcb.2006.08.009.
9. Nagle, J.F. heory of monolayer and bilayer phase transition: effect of headgroup interaction. J. Membr. Biol 1976, 148, 997–1007.
10. Marsh, D. Lateral pressure in membranes. Biochim. Biophys. Acta (BBA)-Rev. Biomembr. 1996, 1286, 183-223. https://doi.org/10.1016/S0304-4157(96)00009-3.
11. Zaborowska, M.; Dziubak, D.; Matyszewska, D.; Sek, S.; Bilewicz, R. Designing a Useful Lipid Raft Model Membrane for Electrochemical and Surface Analytical Studies. Molecules 2021, 26, 5483. https://doi.org/10.3390/molecules26185483.

12. Drolle, E.; Bennett, W.; Hammond, K.; Lyman, E.; Karttunen, M.; Leonenko, Z. Molecular dynamics simulations and Kelvin probe force microscopy to study of cholesterol-induced electrostatic nanodomains in complex lipid mixtures. Soft Matter 2017, 13, 355–362.
13. Henderson, R.D.; Filice, C.T.; Wettig, S.; Leonenko, Z. Kelvin probe force microscopy to study electrostatic interactions of DNA with lipid–gemini surfactant monolayers for gene delivery. Soft Matter 2021, 17, 826–833.
14. Henderson, R.D.E. Nanoscale physics of surfactant gene delivery. PhD dissertation, University of Waterloo, 2016.
15. Nečas, D.; Klapetek, P. Gwyddion– Free SPM (AFM, SNOM/NSOM, STM, MFM, ...) data analysis software. Gwyddion. http://gwyddion.net.
